# Importance of Geospatial Heterogeneity in Chronic Disease Burden for Policy Planning in an Urban Setting Using a Case Study of Singapore

**DOI:** 10.3390/ijerph18094406

**Published:** 2021-04-21

**Authors:** Ken Wei Tan, Joel R. Koo, Jue Tao Lim, Alex R. Cook, Borame L. Dickens

**Affiliations:** Saw Swee Hock School of Public Health, National University of Singapore, National University Health System, Singapore 117549, Singapore; tkwnoel@gmail.com (K.W.T.); ephkoor@nus.edu.sg (J.R.K.); Lim_jue_tao@nea.gov.sg (J.T.L.); ephcar@nus.edu.sg (A.R.C.)

**Keywords:** statistical modeling, chronic disease, spatial epidemiology, urbanization, environmental health

## Abstract

Chronic disease burdens continue to rise in highly dense urban environments where clustering of type II diabetes mellitus, acute myocardial infarction, stroke, or any combination of these three conditions is occurring. Many individuals suffering from these conditions will require longer-term care and access to clinics which specialize in managing their illness. With Singapore as a case study, we utilized census data in an agent-modeling approach at an individual level to estimate prevalence in 2020 and found high-risk clusters with >14,000 type II diabetes mellitus cases and 2000–2500 estimated stroke cases. For comorbidities, 10% of those with type II diabetes mellitus had a past acute myocardial infarction episode, while 6% had a past stroke. The western region of Singapore had the highest number of high-risk individuals at 173,000 with at least one chronic condition, followed by the east at 169,000 and the north with the least at 137,000. Such estimates can assist in healthcare resource planning, which requires these spatial distributions for evidence-based policymaking and to investigate why such heterogeneities exist. The methodologies presented can be utilized within any urban setting where census data exists.

## 1. Introduction

Chronic diseases are the main cause of mortality and morbidity in developed countries, and constitute a large proportion of the healthcare burden [1]. The direct medical costs of various chronic diseases have been estimated to be US$3200–4700 for each type II diabetes mellitus (DM) case annually [2], and US$ 14,000 and US$ 25,000 for each episode of acute myocardial infarction (AMI) and acute ischaemic stroke, respectively [3]. Globally, rising life expectancy has resulted in aging populations such as those in Japan and Taiwan. From 2006 to 2015, the DM prevalence in Japan increased from 12.3% to 19.5% for men and 8.2% to 9.2% for women [4]. For Taiwan, the prevalence of multiple chronic conditions increased from 9.6% to 17.1% from 2000 to 2010 [5]. Singapore, an island city state, experienced an increase in DM prevalence from 8.3% to 8.6% from 2010 to 2017 [6], and from 2007 to 2016, the annual number of AMI and stroke cases increased from 6817 to 10,728 [7] and 5578 to 7413 [8]. As the number of chronic disease cases continues to rise, health systems need to evaluate the accessibility of healthcare facilities and whether they can meet the increase in demand.

To evaluate accessibility and capacity planning, health systems need to identify where cases are in physical space. A method to perform such an analysis would be to use electronic medical records, registries, or other databases that record incident cases [9]. Another way would be to conduct cross-sectional observational studies with the aim of investigating incidence and prevalence [10]. However, there are certain downsides. Electronic medical records only contain information on when an individual made contact with a particular health service which could introduce bias [11]. Multiple regional health systems also have differing diagnostic criteria and capture patient information differently, leading to challenges in linkage to create a single coherent dataset for analysis [12,13,14]. Health data being held by different organizations or institutions further exacerbates the problem of linkage [13]. The same individual who developed two chronic conditions such as AMI and stroke would be entered into different registries or hospital records, making these data sources challenging to work with to answer research questions on comorbidities. Surveys and cross-sectional studies provide a useful single estimate for a point in time, but performing regular cross-sectional studies akin to that of annual population health surveys quickly becomes time and resource intensive [15]. An alternative to answer these research questions is using computational epidemiological methods that provides a quick way to obtain data-driven estimates of incidence or prevalence for research or capacity planning purposes [16].

As computing power improves, computational simulation models are becoming more widely used in public health research. Specifically, chronic diseases develop over a long period of time, and as repeated epidemiological studies are costly and infeasible, computational approaches are useful tools to obtain preliminary estimates, a statement echoed by Barhak et al. [17]. Models such as the Population Health Model in Canada and the United Kingdom Prospective Diabetes Study risk engine are examples of simulation tools that have benefited public health research by providing chronic disease estimates to inform policy [18,19]. For geospatial analyses, a study in New York City developed models from claims data to predict diabetes, hypertension, and asthma prevalence by census tract and visualized the results on a map, creating a view of the geographic distribution of chronic diseases that is easy to understand at a glance [20]. Together, these studies showcase some applications of computational modeling to improve population health.

In this study, we aimed to estimate the present health risk of the resident population of Singapore, an island city state measuring 50 by 27 kilometers, for DM, specifically type II DM, AMI, and stroke using an individual-based model and identify where cases are located geospatially. This model allows us to investigate the presence of comorbidities in the local population quickly without the need to link up patient identifiers between different registries while at the same time provide information as to where these cases are concentrated in physical space. Given that individuals with multiple comorbidities are high-risk and are most likely to seek medical care, we aggregated the number of high-risk individuals by regional health system to generate estimates that are useful for capacity planning in local health institutions.

## 2. Materials and Methods

To perform spatial imputation for a given population, information on the demographic breakdown of the population and the relationship between demographics and geography is required. In this study, we used a temporally explicit agent-based model of the population of Singapore with the demographic attributes of ethnicity, gender, and age by Phan et al. to create a synthetic population of Singapore [21]. This model simulates the population of Singapore from 1990 to 2050 by aging the population year on year, allowing individuals to develop disease such as DM, as well as give birth and die with rates derived from the birth and death registry of Singapore. The population engine was further augmented with disease models of AMI and stroke by Tan et al. to simulate onset of AMI and stroke to create a comprehensive population engine that simulates multiple chronic conditions [22]. As the population engine predicts population health over 60 years, we extracted a cross-section of the population in 2020 to investigate the spatial distribution of chronic disease cases.

The General Household Survey 2015 contains census tables that correlate demographic attributes such as ethnicity, gender and age with physical location [23]. However, a common limitation of censuses with the intention to protect privacy is to obscure individual-level data by publishing anonymized aggregate statistics [24]. This is observed in the General Household Survey where one census table relates physical location to gender and ethnicity while another relates physical location to age. This data was used to recreate a consistent synthetic population across the dimensions of physical location, ethnicity, gender, and age.

A hill climbing algorithm was chosen to optimize across the multiple dimensions. The steps of the algorithm within the context of the study are as follows:

1. Seed random initial physical locations for the synthetic population;
2. Calculate the chi-square statistic for each census table using the synthetic population as the observed values and the census as the expected values;
3. Select two random individuals in the population and swap their physical locations;
4. Calculate the new chi-square statistic for each census table. If the new chi-square statistic is lower than the previous one, accept the swap. If not, reject the swap and revert the physical locations to their original values;
5. Repeat steps 2–4 until improvement in the chi-square statistic is marginal, implying optimal convergence.

This process can be repeated for multiple attributes so long as aggregate statistics relating the imputed attribute with existing attributes in the synthetic population are present. For this study, imputing physical location took 10 million iterations to reach convergence. To obtain robust estimates, we performed spatial imputation with 100 copies of the synthetic population and reported the means.

Using this method, specific outcomes of interest were chosen for validation and exploratory purposes. The National Health Survey 2010 is a population health survey for Singaporean citizens and permanent residents aged 18–69. In the National Health Survey 2010, the prevalence of DM was reported by ethnicity and gender in one table and by age group. We recreated these tables using the synthetic population for 2020, reporting the prevalence and the expected number of cases for validation purposes, and did the same for the chronic conditions of AMI and stroke where there are currently limited reported estimates at a population level. Registry data only reports annual incident cases which is insufficient to estimate the total chronic disease burden in the entire population. To estimate prevalence, a temporal element is required, hence our modelling approach. With a cross-section of the population in 2020, we can then obtain prevalent cases in the community. Singapore aggregates population statistics by planning areas, so we aggregated and visualized these case numbers on a map at the planning area level as a simple way to quickly assess the spatial distribution of health. Additionally, this model consolidates all health conditions by individual and allows for detailed reporting of comorbidities at a population level while simultaneously identifying where these high-risk individuals are likely to be located. To understand the spatial distribution of comorbidities, we investigated the number of individuals with DM and AMI, DM and stroke, AMI and stroke as well as all three chronic conditions and visualized the results on a map of Singapore. 

In Singapore, healthcare providers are organized into three regional health systems in the western, northern, and eastern parts of Singapore, thus for capacity planning, we aggregated our results by health system, a superset of physical location. We presented the total number of high-risk individuals with at least one chronic condition in each of the three regional health systems as these individuals are most likely to seek medical care for chronic conditions.

## 3. Results

Table 1 provides estimates of the number and prevalence of DM, AMI, and stroke cases in the Singapore population aged 18–69 in 2020. In 2010, the National Health Survey reported overall DM prevalence of 11.3%. Females show significantly lower susceptibility to DM in contrast with males [25], with 10.1% of females and 15.3% of males having DM. Ethnicity is also a big risk factor with Malays (males: 20.0%, females: 17.1%) and Indians (males: 23.2%, females: 20.3%) showing significantly higher prevalence of DM as compared to their Chinese counterparts (males: 13.2%, females: 7.8%) regardless of gender, echoing previous results by Phan et al. and the National Health Survey 2010 stating that the burden of DM would be borne disproportionately [21,26]. This susceptibility to DM translates to higher prevalence in males for both AMI and stroke (AMI: 5.0%, stroke: 2.5%) as compared to females (AMI: 0.9%, stroke: 1.3%) with about seven times more males with past AMI and two times more with stroke. These results are consistent with annual reports from the Singapore Myocardial Infarction Registry and the Singapore Stroke Registry which reported from 2008 to 2017 that males had incidence rates approximately twice that of females for AMI and 1.3 times that of females for stroke [27,28]. Although there are no published estimates of past AMI prevalence to validate our estimate of 3.0% in Singapore, the prevalence of stroke was 3.65% across the entire population in 2006 [29], suggesting that our estimate for those aged 18–69 of 1.9% is plausible. 

After stratifying by age (Table 2), we observe a standard dose response relationship with age and disease prevalence. When comparing DM prevalence with the National Health Survey 2010 [26], all age groups have an absolute deviation of less than 1%, ranging from the youngest age group of 18–29 (actual: 1.0%, estimated: 1.5%) to the oldest at 60–69 (actual: 29.1%, estimated: 29.8%). 

**Figure 1:**
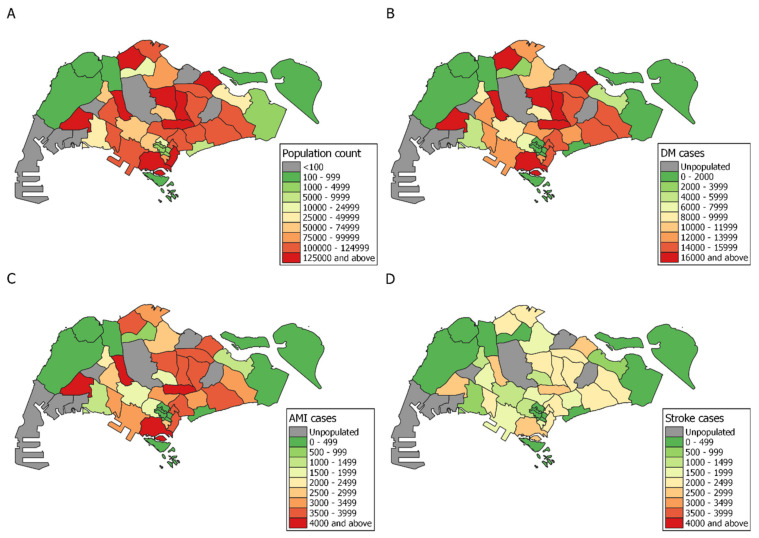
National-level maps of (**A**) population count (**B**) diabetes mellitus (DM) (**C**) acute myocardial infarction (AMI) and (**D**) stroke case numbers aggregated by planning area. Areas with less than 100 residents were defined as unpopulated and are coloured gray. Simpang and Seletar Island have been omitted as fewer than 10 individuals reside in these locations.

Figure 1 depicts the population count and chronic disease cases for planning areas with at least 100 individuals. Number of DM cases tends to correlate with population density, but although DM prevalence in Singapore averages at about 12.7%, it can be observed that prevalence varies by area, ranging from 12.5% to 12.9% (Table A1). AMI and stroke follow the same pattern at 3.0% and 1.9%, ranging from 2.9% to 3.2% for AMI and 1.8% to 2.1% for stroke. In terms of absolute case numbers, due to higher population density, we observe high-risk clusters with >14,000 DM cases per planning area in the north-eastern area of Singapore, indicating high chronic disease burden. As DM is a major risk factor for other chronic diseases, it is reasonable to assume that this group of people are highly susceptible to developing comorbidities, if they have not done so already. AMI has comparatively fewer cases, with the same cluster in the northeast having approximately 4000 past AMI cases per planning area. Risk of developing a second recurrent AMI increases gradually over time, hence this group requires a quick response time and admittance to the accident and emergency departments to prevent fatalities [30]. Stroke displays a more even spatial distribution, with most planning areas having stroke cases in the 2000–2500 range. These prevalent stroke cases require extensive psychosocial support and rehabilitation post-stroke [31]. Therefore, it is prudent to situate psychological services and outlets for physiotherapy in the vicinity for their benefit.

**Figure 2:**
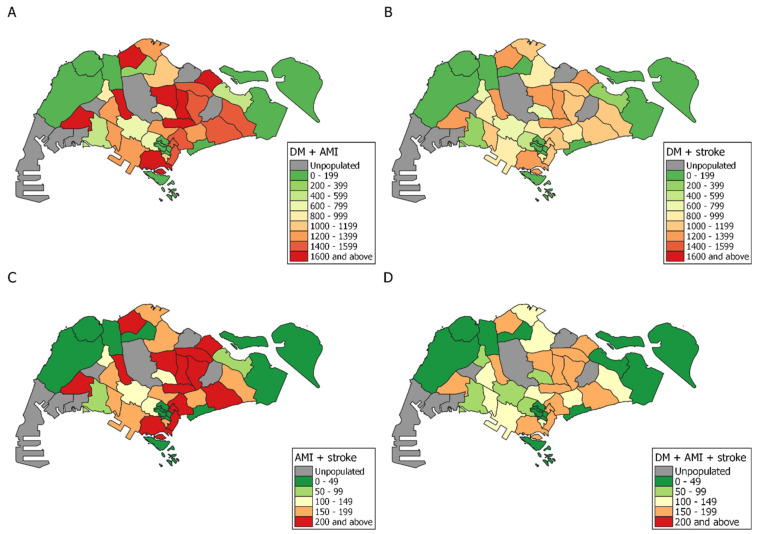
National level maps of comorbidities aggregated by planning area. (**A**) diabetes mellitus (DM) and acute myocardial infarction (AMI), (**B**) DM and stroke, (**C**) AMI and stroke, (**D**) DM, AMI, and stroke. Areas with less than 100 residents were defined as unpopulated and are coloured gray. Simpang and Seletar Island have been omitted as fewer than 10 individuals reside in these locations.

Beyond the scope of estimating prevalence of individual conditions, we also investigated comorbidities (Figure 2). DM with AMI was the most common combination of chronic conditions as DM is a major biological risk factor for AMI [32], with approximately 10% of all individuals with DM also having AMI. DM and stroke is the second most common combination, with 6% of all individuals with DM having stroke concurrently. Panel C of Figure 2 indicates that AMI and stroke is not a common combination, as there are at most around 200 individuals with this combination per planning area, reinforcing that DM is a precursor to AMI and stroke. Lastly, for all three conditions, there are at most 150–199 individuals per planning area. Most of these planning areas are also in the northeast, supporting the notion that this area is in greatest need of healthcare amenities. This group also represents those who have the highest mortality rates, prompting the need for interventions to improve nutrition and physical activity in an effort to reduce mortality.

**Figure 3:**
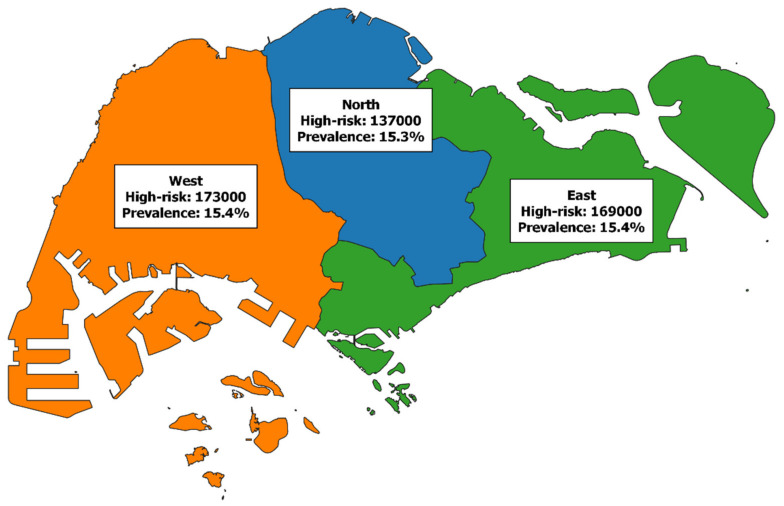
High-risk cases and prevalence by regional health system, rounded off to the nearest thousand. High-risk cases were defined as individuals with at least one chronic disease.

The other application of the model is to visualize aggregate data which would otherwise require some coordination to obtain across different healthcare providers. Figure 3 illustrates this by presenting the number of individuals with at least one chronic disease, assumed to be high-risk and therefore requiring health services, by regional health system. Of the three different regional health systems, each of them has similar prevalence ranging from 15.3% to 15.4%. The similar prevalence indicates that the disparity in case numbers is driven by population density. The western region is the most populated and therefore has the greatest number of high-risk individuals at 173,000. This is followed by the eastern region with 169,000 and the smallest northern region at 137,000. These metrics provide valuable insight for strategic planning teams in each of the regional healthcare hubs to assess whether their current healthcare facilities are adequate to sustain these at-risk individuals. 

## 4. Discussion

Applying our model to the population of Singapore, we obtained quick estimates of the case numbers and prevalence of DM, AMI, and stroke in Singapore. Our results suggest that prevalence varies greatly by age, gender, and ethnicity, reinforcing previous findings that in Singapore, disease risk is heavily influenced by demographics [33]. These estimated prevalence values are further validated by being very similar to the reported values in the National Health Survey. These similar age-specific prevalence values across a decade suggest that risk within the population is not inherently changing. For additional validation, an independent study of 2562 participants of which 1415 were aged 60–74, reported a stroke prevalence of 5.7% which was coherent with our estimate of stroke prevalence in those aged 60–69 at also 5.7% [34]. Of the three conditions, AMI and stroke were comparatively harder to obtain prevalence estimates as compared to DM due to high mortality rates, especially in the first year following the event [27,28]. Furthermore, unlike DM, a single individual can have multiple AMIs and strokes over the course of their lifetime and is therefore in a constant high-risk state as the conditions can recur, influenced by age and whether an individual also has DM [35]. Timely access to healthcare is critical for this subgroup of people who stand to lose more than the average healthcare consumer should emergency departments be over capacity or have long waiting times [36]. The national-level maps provide useful insights into these high-risk communities, which is useful for evidence-driven policy making and capacity planning [37,38]. From a health services research perspective, these maps of chronic disease risk aid urban planners and regional health systems in ensuring that the facilities in an area are sufficient to support the healthcare needs of the community [39]. The maps presented are cross-sectional but indicate where hotspots of chronic disease could arise as a result of an aging population, assuming no significant changes in inter-planning area migration patterns. Figure 3 and Figure 2 together reiterate that access to healthcare is most required in the western region and the cusp of the north and eastern region where we estimate the largest numbers of at-risk individuals are located. From 2009 to 2017, the proportion of those aged 60 and above suffering from three or more chronic conditions increased from 19.8% to 37.0% [40], reflecting the growing chronic disease burden and prompting the need to maintain access to adequate medical care.

Apart from access to timely medical care, urban planners choosing potential locations of homes for the elderly, dialysis centers, or other primary care facilities would benefit from information on the spatial distribution of chronic disease risk. Healthy Urban Planning is one of the themes of the World Health Organization [41]; the objectives of this theme are to promote healthy lifestyles, facilitate access to healthy food, and increase accessibility to healthcare facilities through proper urban planning. Healthy Urban Planning initiatives can make use of maps of chronic disease risk in order to improve population health through urban design. At a population level, urban interventions allowing for greater access to healthcare and healthier lifestyle options have a small effect at the individual-level, but can potentially have a large cumulative effect across the whole population [42].

Globally, there is increased awareness as to the connection between urban planning and the health of communities [43]. Our method was applied to Singapore but is applicable to other populations to estimate the geospatial distribution of chronic disease to inform health policy. A case study of Dortmund, Germany, identified significant associations between green space, air quality, and socioeconomically disadvantaged communities [44]. In China, a link between population density and obesity was reported based on data from 450 communities over 30 provinces [45]. At the national level, the Canadian Urban Environmental Health Research Consortium was established in 2015 to consolidate a wealth of geospatial exposure data and cohort studies for healthcare research, including features such as transportation networks and land use [46]. All these examples illustrate that the appropriate combination of census and geospatial information have been used to generate geospatial distributions of risk factors and diseases. The goal is therefore to formulate evidence-driven policy at the city scale that many planners operate in [38,47]. As the link between urban planning and public health continues to develop, geospatial modelling of chronic diseases becomes more useful in deriving insights.

Potential improvements to this study would be obtaining the actual anonymized geographic distribution of the population with detailed health information as opposed to a synthetically reconstructed population for additional validation, although this was not possible due to issues of data privacy. We also were not able to compare prevalence of AMI and stroke with actual numbers as registry reports primarily report incidence, and the last estimate of stroke prevalence was in 2006. In the evaluation of access to hospitals and healthcare facilities, it is common to define catchment areas based on geography [48]; however, in practice numerous other factors may influence where an individual chooses to seek medical care such as their immediate location, access to transport or personal preference.

## 5. Conclusions

Geographical distributions of chronic disease risk are instrumental in understanding differences in community health at a national level and identifying high-risk communities which is critical for evidence-based policymaking. Using Singapore as a case study, we estimated the prevalence of DM, AMI, and stroke to be 12.7%, 3.0%, and 1.9%, respectively. In terms of comorbidities, approximately 10.0% and 6.0% of individuals with DM had AMI. To inform urban planning, we provided estimates of the number of high-risk cases by regional health system in order to ensure adequate access to healthcare and to minimise geographical disparities in health outcomes. 

## Figures and Tables

**Table 1:** Estimated case numbers and prevalence of Diabetes Mellitus (DM), past acute myocardial infarction (AMI) and stroke for those aged 18–69 in 2020 stratified by ethnicity and gender. Brackets indicate the 95% confidence intervals over 100 iterations.

Demographic Group	DM Cases (thousands)	AMI Cases (thousands)	Stroke Cases (thousands)	DM Prevalence	Past AMI Prevalence	Stroke Prevalence
Chinese males	152.3 (151.7, 153.0)	41.2 (40.8, 41.5)	28.0 (27.7, 28.4)	13.2 (13.2, 13.3)	3.6 (3.5, 3.6)	2.4 (2.4, 2.5)
Chinese females	87.6 (87.0, 88.1)	6.2 (6.1, 6.3)	12.9 (12.6, 13.1)	7.8 (7.7, 7.8)	0.6 (0.5, 0.6)	1.1 (1.1, 1.2)
Malay males	38.8 (38.5, 39.1)	18.1 (17.8, 18.3)	7.2 (7.0, 7.3)	20.0 (19.9, 20.2)	9.3 (9.2, 9.5)	3.7 (3.6, 3.8)
Malay females	32.6 (32.3, 33.0)	3.9 (3.8, 4.0)	4.1 (4.0, 4.3)	17.1 (16.9, 17.2)	2.0 (2.0, 2.1)	2.2 (2.1, 2.2)
Indian males	43.6 (43.2, 43.9)	15.5 (15.2, 15.7)	3.9 (3.8, 4.1)	23.2 (23.1, 23.4)	8.3 (8.1, 8.4)	2.1 (2.1, 2.2)
Indian females	29.9 (29.6, 30.2)	2.9 (2.8, 3.0)	1.8 (1.8, 1.9)	20.3 (20.2, 20.5)	2.0 (1.9, 2.0)	1.3 (1.2, 1.3)
Other males	8.2 (8.0, 8.4)	4.9 (4.8, 5.0)	1.1 (1.0, 1.1)	14.1 (13.9, 14.5)	8.5 (8.3, 8.7)	1.9 (1.7, 2.0)
Other females	3.9 (3.7, 4.0)	0.6 (0.6, 0.7)	0.4 (0.4, 0.5)	6.7 (6.5, 6.9)	1.1 (1.0, 1.2)	0.8 (0.7, 0.8)

**Table 2:** Estimated case numbers and prevalence of DM, past AMI and stroke for those aged 18–69 in 2020 stratified by age group. Brackets indicate the 95% confidence intervals over 100 iterations.

Age Group	DM Cases (thousands)	AMI Cases (thousands)	Stroke Cases (thousands)	DM Prevalence	Past AMI Prevalence	Stroke Prevalence
18–29	10.2 (10.0, 10.4)	2.8 (2.7, 2.9)	1.6 (1.5, 1.7)	1.5 (1.5, 1.5)	0.4 (0.4, 0.4)	0.2 (0.2, 0.2)
30–39	34.4 (34.1, 34.7)	7.7 (7.6, 7.9)	4.2 (4.0, 4.3)	5.3 (5.3, 5.4)	1.2 (1.2, 1.2)	0.7 (0.6, 0.7)
40–49	73.0 (72.6, 73.4)	13.7 (13.5, 13.9)	7.6 (7.4, 7.8)	11.3 (11.3, 11.4)	2.1 (2.1, 2.2)	1.2 (1.2, 1.2)
50–59	124.5 (124.0, 125.0)	27.3 (26.9, 27.6)	16.7 (16.5, 16.9)	19.9 (19.8, 20.0)	4.4 (4.3, 4.4)	2.7 (2.6, 2.7)
60–69	154.8 (154.2, 155.4)	41.7 (41.4, 42.0)	29.5 (29.1, 29.8)	29.8 (29.6, 29.9)	8.0 (8.0, 8.1)	5.7 (5.6, 5.7)

## Data Availability

Data from the General Household Survey 2015 is publicly available at https://www.singstat.gov.sg/publications/ghs/ghs2015. Last accessed on 1 March 2021.

## Appendix A

**Table A1:** Population count, absolute case numbers, and prevalence for DM, AMI, and stroke by planning area.

Planning Area	DM Cases	AMI Cases	Stroke Cases	DM Prevalence (%)	AMI Prevalence (%)	Stroke Prevalence (%)	Population
ANG MO KIO	16,144	3807	2424	12.68	2.97	1.91	127,072
BEDOK	15,692	3705	2341	12.85	3.02	1.91	122,710
BISHAN	8365	1954	1261	12.79	3.03	1.91	65,059
BOON LAY	6	2	1	19.31	5.16	1.44	40
BUKIT BATOK	12,753	2962	1930	12.87	3	1.95	100,088
BUKIT MERAH	18,123	4274	2709	12.79	3	1.92	141,752
BUKIT PANJANG	18,412	4372	2744	12.64	2.98	1.88	144,840
BUKIT TIMAH	8029	1871	1208	12.96	3.03	1.94	62,103
CENTRAL WATER CATCHMENT	3	1	0	15.78	5.09	1.99	21
CHANGI	263	62	37	12.49	2.98	1.76	2083
CHOA CHU KANG	8890	2067	1338	12.81	2.97	1.92	70,095
CLEMENTI	13,738	3256	2052	12.82	3	1.9	106,644
DOWNTOWN CORE	15,890	3717	2384	12.63	2.94	1.9	125,886
GEYLANG	12,921	3020	1942	12.88	3	1.94	100,403
HOUGANG	15,640	3670	2361	12.72	2.96	1.92	122,791
JURONG EAST	4911	1147	742	12.91	3.04	1.93	38,102
JURONG WEST	17,695	4163	2650	12.54	2.92	1.89	140,248
KALLANG	15,593	3650	2338	12.76	2.98	1.92	122,948
LIM CHU KANG	41	10	6	14.21	3.99	1.85	323
MANDAI	2386	561	355	12.89	3.08	1.86	18,524
MARINE PARADE	1067	253	156	12.82	3.05	1.86	8218
MUSEUM	540	130	75	12.38	2.92	1.72	4300
NEWTON	1489	352	220	13.08	3.13	1.88	11,378
NORTH-EASTERN ISLANDS	19	5	2	16.79	4.63	2.05	138
NOVENA	13,007	3030	1954	12.51	2.9	1.87	103,267
ORCHARD	173	42	24	12.66	3.05	1.71	1389
OUTRAM	12,003	2812	1797	12.87	3.01	1.93	93,343
PASIR RIS	4341	1008	654	12.85	3.04	1.88	33,857
PAYA LEBAR	9	2	1	14.47	4.22	2	62
PIONEER	9	3	1	16.01	4.72	2.44	62
PUNGGOL	16,953	3993	2546	12.69	2.96	1.91	132,833
QUEENSTOWN	13,008	3027	1965	12.64	2.93	1.9	102,433
RIVER VALLEY	232	56	33	12.58	3.16	1.74	1858
ROCHOR	15,692	3690	2364	12.74	2.98	1.92	123,289
SELETAR	10	2	1	14.79	4.29	2.12	66
SEMBAWANG	13,519	3170	2030	12.63	2.95	1.9	107,035
SENGKANG	15,015	3550	2256	12.89	3.03	1.94	116,560
SERANGOON	16,727	3947	2488	12.68	2.97	1.89	131,887
SINGAPORE RIVER	394	96	57	12.2	2.86	1.74	3172
SOUTHERN ISLANDS	103	25	14	12.76	3.11	1.68	832
SUNGEI KADUT	87	21	12	13.87	3.27	1.91	692
TAMPINES	14,065	3318	2108	12.76	3	1.92	110,510
TANGLIN	7460	1732	1126	12.83	2.98	1.93	58,295
TENGAH	11	2	1	16.2	3.71	2.1	67
TOA PAYOH	17,297	4071	2577	12.41	2.9	1.85	138,303
TUAS	9	2	1	15.45	4.13	1.93	60
WESTERN WATER CATCHMENT	103	25	14	12.89	3.12	1.76	809
WOODLANDS	16,492	3906	2472	12.7	2.98	1.91	129,993
